# Arthralgia among women taking aromatase inhibitors: is there a shared inflammatory mechanism with co-morbid fatigue and insomnia?

**DOI:** 10.1186/s13058-015-0599-7

**Published:** 2015-06-28

**Authors:** Joshua Bauml, Lu Chen, Jinbo Chen, Jean Boyer, Michael Kalos, Susan Q. Li, Angela DeMichele, Jun J. Mao

**Affiliations:** Abramson Cancer Center, University of Pennsylvania, Philadelphia, PA USA; Department of Medicine, Division of Hematology/Oncology, Perelman School of Medicine, University of Pennsylvania, Philadelphia, PA USA; Center for Clinical Epidemiology and Biostatistics and Department of Biostatistics and Epidemiology, Perelman School of Medicine, University of Pennsylvania, Philadelphia, PA USA; Department of Pathology and Laboratory Medicine, Perelman School of Medicine, University of Pennsylvania, Philadelphia, PA USA; Eli Lilly & Company, New Hyde Park, NY USA; Department of Family Medicine and Community Health, Perelman School of Medicine, University of Pennsylvania, 3400 Spruce Street, 2 Gates, Philadelphia, PA 19104 USA

## Abstract

**Introduction:**

Arthralgia is a common toxicity among women taking aromatase inhibitors (AIs) and can lead to premature discontinuation of therapy. We evaluated the association between arthralgia, co-morbid fatigue and/or insomnia, and inflammatory biomarkers among women taking AIs.

**Methods:**

Women taking AIs for early-stage breast cancer completed a modified version of the Brief Pain Inventory, the Brief Fatigue Inventory, and the Insomnia Severity Index and provided blood samples for simultaneous assessment of 34 inflammatory biomarkers with a Luminex kit. Two-sided *t* tests were used to compare inflammatory biomarker concentrations for patients with or without moderate to severe arthralgia. Multivariate linear regression analyses were performed to evaluate the relationship between comorbid arthralgia, fatigue, and insomnia with identified biomarker concentrations.

**Results:**

Among 203 participants, the severity of arthralgia, fatigue, and insomnia were significantly correlated with each other (*p* < 0.001 for all comparisons). After controlling for race, chemotherapy history, non-steroidal anti-inflammatory drug use, age, and body mass index, the coexistence of arthralgia, fatigue, and insomnia was associated with elevated C-reactive protein (CRP) (β = 93.1; 95 % confidence interval (CI): 25.1–161.1; *p* = 0.008), eotaxin (β = 79.9; 95 % CI: 32.5–127.2; *p* = 0.001), monocyte chemoattractant protein (MCP)-1 (β = 151.2; 95 % CI: 32.7–269.8; *p* = 0.013), and vitamin D–binding protein (VDBP) (β = 19,422; 95 % CI: 5500.5–33,344; *p* = 0.006).

**Conclusions:**

Among women taking AIs, the coexistence of arthralgia, fatigue, and insomnia was associated with increased levels of inflammatory biomarkers (elevated CRP, eotaxin, MCP-1, and VDBP). These findings suggest a possible shared inflammatory mechanism underlying these common symptoms.

**Electronic supplementary material:**

The online version of this article (doi:10.1186/s13058-015-0599-7) contains supplementary material, which is available to authorized users.

## Introduction

Aromatase inhibitors (AIs) are an important component of the standard treatment of hormone receptor–positive breast cancer, but a high incidence of arthralgia (joint pain) may lead to premature therapy discontinuation and increased mortality [1, 2]. Although up to 47 % of women taking AIs report arthralgia [3], current understanding of the mechanisms underlying AI-associated arthralgia is limited.

Menopause and other estrogen withdrawal syndromes are associated with an increase is systemic inflammation, which can exacerbate joint pain syndromes [4–8]. Given the precipitous drop in estrogen levels associated with AI administration [9], AI-associated arthralgia could be a result of similar immunomodulation. Imaging studies have revealed inflammatory tenosynovial changes at sites of AI-associated arthralgia, and a genome-wide association study revealed four single-nucleotide polymorphisms (SNPs) linking AI-associated arthralgia and T-cell maturation [10, 11]. Prior efforts to evaluate the association between AI-associated arthralgia and systemic inflammation have been limited by small sample sizes [12, 13]. In contrast, fatigue and insomnia, which are often coexistent with pain in patients with cancer [14], have been associated with systemic inflammation [15–17]. In fact, the coexistence of arthralgia, fatigue, and insomnia is one of the more common symptom clusters experienced by breast cancer survivors [18, 19].

We aimed to evaluate the association of moderate to severe arthralgia with plasma inflammatory biomarker concentrations among postmenopausal women with early-stage breast cancer who were currently taking AIs. We hypothesized that the presence of moderate to severe arthralgia would be associated with elevated serum inflammatory biomarker concentrations. Because arthralgia often coexists with fatigue and insomnia [19], we then evaluated the association of the simultaneous experience of arthralgia, fatigue, and insomnia with inflammatory biomarkers. We hypothesized that the molecules identified in aim 1 would also be positively correlated with the coexistence of arthralgia, fatigue, and insomnia.

## Methods

### Study design and patients

We conducted a cross-sectional study, drawing our sample from an ongoing cohort study of patients receiving adjuvant AI therapy in the outpatient breast oncology clinic in the Abramson Cancer Center at the University of Pennsylvania between 2008 and 2013. Eligible participants were aged 18 years or older, had a diagnosis of early-stage breast cancer (stages I–III), were taking an AI, and had a Karnofsky score ≥60 (i.e., ambulatory). Additional inclusion criteria required the completion of all chemotherapy and radiation therapy at least 1 month before enrollment, approval of the patient’s treating oncologist, and the ability of the patient to understand and provide informed consent in English. After providing informed consent, patients completed a series of patient-reported outcome instruments and provided a non-fasting blood sample for analysis. Self-report questionnaires and chart abstraction were used for demographic and clinical information. For the present study, we included 203 participants who had had both survey data and a blood sample available during the time of biomarker analyses. The Institutional Review Board at the University of Pennsylvania approved this study.

### Symptom measurement

Arthralgia was measured using a modified version of the Brief Pain Inventory (BPI). Because we were focused on arthralgia, the questions were modified to evaluate pain in and around joints. The BPI is validated in patients with cancer and is one of the most commonly used instruments used to measure pain. Domains measured are pain severity (four items) and interference (seven items), with Cronbach’s α ranging from 0.8 to 0.87 and from 0.89 to 0.92, respectively [20]. The use of a single item on the instrument, “Worst pain in the last 24 hours,” has previously been validated for use of the BPI as a dichotomous measure to define moderate to severe pain [21]. Our group has previously shown that a cut point ≥4 on this item was associated with greater likelihood to discontinue AI therapy prematurely [1].

Fatigue was measured using the Brief Fatigue Inventory (BFI). The BFI has been validated in cancer populations (Cronbach’s α = 0.96). Similar to the BPI, the “worst fatigue” item has been validated as a single-item dichotomous variable, with a cut point ≥4 indicating moderate to severe fatigue [22]. Insomnia was measured using the Insomnia Severity Index, a seven-item survey validated in the assessment of insomnia severity among patients with cancer with a Cronbach’s α ranging from 0.76 to 0.78. A cut point >14 on the overall score has been validated as a dichotomous measure for the presence or absence of moderate to severe insomnia [23].

### Inflammatory biomarker measurement

Serum levels of cytokines, chemokines, and inflammatory molecules were determined using the Cytokine Human Magnetic 30-Plex Panel for the Luminex platform (Life Technologies, Carlsbad, CA, USA) and the Acute Phase Human 4-Plex Panel for the Luminex platform (Life Technologies). With the 30-plex assay, we examined the following serum cytokine and chemokine levels: interleukin (IL)-1β, IL-1RA, IL-2, IL-2R, IL-4, IL-5, IL-6, IL-7, IL-8, IL-10, IL-12p40/p70, IL-13, IL-15, IL-17, vascular endothelial growth factor, tumor necrosis factor α, interferon (IFN)-α, IFN-γ, granulocyte macrophage colony-stimulating factor, macrophage inflammatory protein (MIP)-1α, MIP1-β, IFN-γ-inducible protein 10, monokine induced by interferon gamma, eotaxin, the regulated on activation, normal T expressed and secreted protein (RANTES), monocyte chemoattractant protein (MCP)-1, epidermal growth factor, granulocyte colony-stimulating factor, basic fibroblast growth factor, and hepatocyte growth factor. Sera were defrosted on ice and added undiluted to the beads. Incubation and washing were carried out per assay specifications. With the 4-plex acute phase panel, we examined β_2_-microglobulin, C-reactive protein (CRP), haptoglobin, and vitamin D–binding protein (VDBP or Gc globulin). Sera were diluted as suggested to a 1:8000 concentration. The diluted sample was added to the magnetic beads, followed by incubation and washing per assay specifications.

### Statistical analysis

Descriptive statistics were used to evaluate the distribution of scores on each of the three patient-reported outcome instruments, with which we measured arthralgia, fatigue, and insomnia. Patients were then dichotomized into the presence or absence of moderate to severe arthralgia, fatigue, and insomnia, as defined by the cut points described above. We performed χ^2^ analysis to assess pairwise correlations among the three symptoms.

We explored the variability in each inflammatory biomarker using box plots. We identified 20 biomarkers with limited intersubject variability. These were excluded from further analyses. We assessed pairwise correlations among the 14 remaining biomarkers, which revealed a significant association. To understand whether the variability in inflammatory biomarkers could be explained by clinical and demographic variables, we performed a series of linear regressions to evaluate the association of our collected clinical and demographic variables with inflammatory biomarker concentration. With our identified inflammatory biomarkers, we specifically assessed the association of each cytokine marker with age, race, non-steroidal anti-inflammatory drug (NSAID) use in the last 7 days, body mass index (BMI), education, years since last menstrual period, history of chemotherapy administration (with a specific focus on taxane history, given the risk for acute taxane induced neuropathy, which can present as joint pain), cancer stage, and years on AIs. Any variable with a *p* value ≤0.1 was included in our final model.

For the primary hypothesis, we conducted two-sided *t* tests to compare the mean concentration of each inflammatory biomarker for patients with and without moderate to severe arthralgia. The Bonferroni adjustment was applied to adjust for multiplicity in testing (*p* < 0.004). For the secondary hypothesis, we created a composite binary variable to identify patients with all three symptoms versus those with fewer symptoms. We then conducted two-sided *t* tests to compare the mean concentration of each inflammatory biomarker for patients with and without comorbid symptoms. Finally, we performed multivariate linear regression analysis to reevaluate the associations between symptoms and inflammatory biomarkers after adjusting for confounding variables.

## Results

### Patient characteristics

The demographic and clinical characteristics of study participants are shown in Table 1. Our analysis included 203 patients. Their mean age was 60.5 years, and 80.7 % of them were white. The stage distribution was as follows: 47 % in stage 0/I, 40.5 % in stage II, and 12.5 % in stage III. The most common AI used was anastrozole, which was used by 74.4 % of participants. Chemotherapy had been administered to 61.7 % of patients surveyed.

**Table 1:** Participant characteristics (*N* = 203)

	Overall population	With symptom cluster	Without symptom cluster
Mean age (SD)	60.5 (8.5) yr	60.1 (9.4) yr	60.6 (8.3) yr
Body mass index (kg/m^2^)			
Normal weight (18.5–24.9)	83 (41.1 %)	9 (26.5 %)	74 (44 %)
Overweight (25–29.9)	59 (29.2 %)	12 (35.3 %)	47 (28 %)
Obese (>30)	60 (29.7 %)	13 (38.2 %)	47 (28 %)
Race			
White	163 (80.7 %)	26 (76.5 %)	137 (81.5 %)
Non-white	39 (19.3 %)	8 (23.5 %)	31 (18.5 %)
Education			
High school or less	46 (22.8 %)	8 (23.5 %)	38 (22.6 %)
College	93 (46 %)	20 (58.8 %)	73 (43.5 %)
Graduate/professional	63 (31.2 %)	6 (17.7 %)	57 (33.9 %)
Years since LMP			
<5 yr	41 (21.5 %)	13 (40.6 %)	80 (50.3 %)
5–10 yr	57 (29.8 %)	10 (31.3 %)	47 (29.6 %)
>10 yr	93 (48.7 %)	9 (28.1 %)	32 (20.1 %)
Cancer stage			
Stage 0/I	94 (47 %)	13 (38.2 %)	81 (48.8 %)
Stage II	81 (40.5 %)	16 (47.1 %)	65 (39.2 %)
Stage III	25 (12.5 %)	5 (14.7 %)	20 (12 %)
AI taken			
Anastrozole	148 (74.4 %)	27 (79.4 %)	121 (73.3 %)
Exemestane	20 (10 %)	2 (5.9 %)	18 (10.9 %)
Letrozole	31 (15.6 %)	5 (14.7 %)	26 (15.8 %)
Years on AI			
<1 yr	54 (26.7 %)	11 (32.4 %)	43 (25.6 %)
1–3 yr	85 (42.1 %)	12 (35.3 %)	73 (43.5 %)
>3 yr	63 (31.2 %)	11 (32.3 %)	52 (30.9 %)
Chemotherapy history			
None	77 (38.3 %)	14 (41.2 %)	63 (37.7 %)
Regimen with a taxane	78 (38.8 %)	10 (29.4 %)	36 (21.6 %)
Regimen without a taxane	46 (22.9 %)	10 (29.4 %)	68 (40.7 %)
NSAID use in last 7 days			
Yes	61 (30.3 %)	17 (50 %)	44 (26.3 %)
No	138 (68.7 %)	17 (50 %)	121 (72.5 %)
Don’t know	2 (1 %)	0 (0 %)	2 (1.2 %)

*AI* aromatase inhibitor, *LMP* last menstrual period, *NSAID* non-steroidal anti-inflammatory drug, *SD* standard deviation

*Symptom cluster* refers to comorbid arthralgia, fatigue, and insomnia

### Prevalence and correlation of key symptoms

Using the cut points of moderate to severe symptoms defined above, 21.3 % of patients had arthralgia, 41.6 % of patients had fatigue, and 33.2 % had insomnia. Arthralgia was significantly correlated with fatigue (*r* = 0.56, *p* < 0.001), and 88.4 % of patients with arthralgia also had fatigue. Arthralgia was also significantly correlated with insomnia (*r* = 0.49, *p* < 0.001), and 83.7 % of patients with arthralgia also had insomnia. Of the overall population, 16.8 % of patients had all three symptoms. The demographic and clinical characteristics of study participants, as a function of the presence or absence of all three symptoms, are shown in Table 1.

### Association between arthralgia, fatigue, and insomnia with biomarkers

**Fig. 1:**
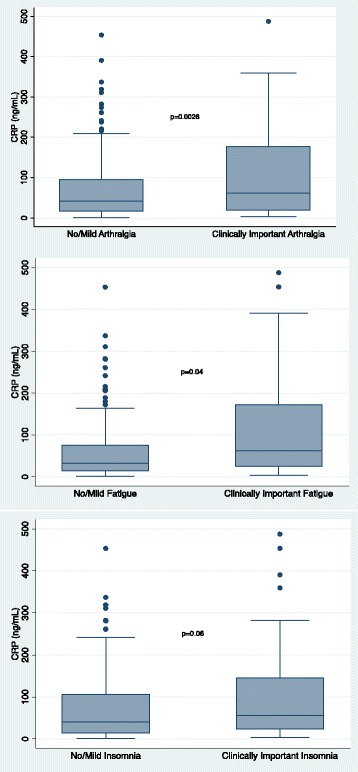
Association of individual symptoms with C-reactive protein (CRP)

**Fig. 2:**
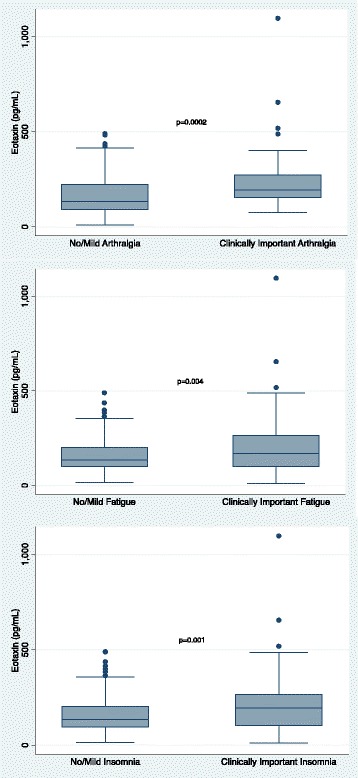
Association of individual symptoms with eotaxin

**Fig. 3:**
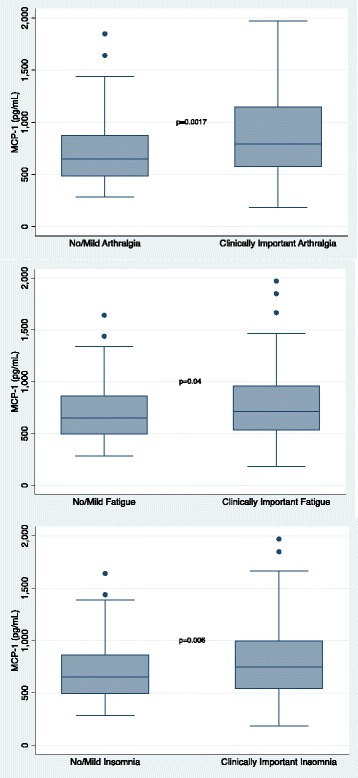
Association of individual symptoms with monocyte chemoattractant protein (MCP)-1

**Fig. 4:**
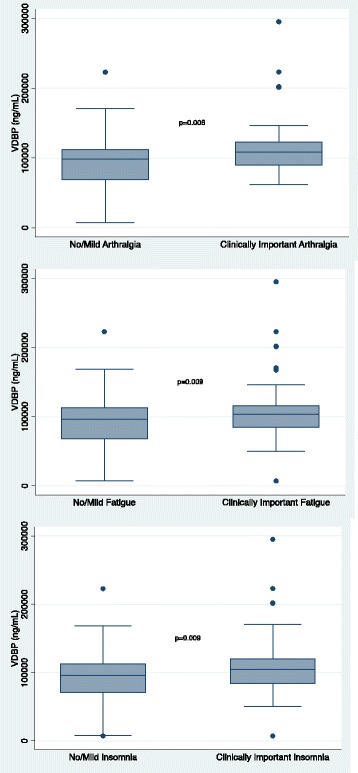
Association of individual symptoms with vitamin D–binding protein (VDBP)

The biomarkers exhibited significant pairwise correlation (Additional file 1: Table S1). As such, a multivariable model including all inflammatory biomarkers was not constructed. As can be seen in Figs. 1, 2, 3, and 4, higher concentrations of CRP (*p* = 0.0026), eotaxin (*p* = 0.0002), MCP-1 (*p* = 0.0017), and VDBP (*p* = 0.0006) were associated with moderate to severe arthralgia. These results were all statistically significant after correction for multiple comparisons. In an exploratory analysis of fatigue and insomnia, all of these biomarkers also showed an association with fatigue and insomnia, with the exception of a trend toward significance for CRP and insomnia. Additional file 2: Tables S2–S4 describe the results of all two-sided *t* tests done to evaluate the association of each symptom with inflammatory biomarkers.

### Association of composite symptom variable with inflammatory biomarkers

We performed a series of linear regressions to evaluate the association of our composite symptom burden variable with biomarker concentrations, controlling for BMI, race, chemotherapy status, NSAID use, and age. BMI, NSAID use, and race were associated with inflammatory biomarker concentrations in our sample (data not shown), whereas chemotherapy and age have been shown in other series to impact arthralgia and inflammatory biomarker concentrations [15, 24, 25]. CRP (β = 93.1; 95 % confidence interval [CI]: 25.1–161.1; *p* = 0.008), eotaxin (β = 79.9; 95 % CI: 32.5–127.2; *p* = 0.001), MCP-1 (β = 151.2; 95 % CI: 32.7–269.8; *p* = 0.013), and VDBP (β = 19,422; 95 % CI: 5500.5–33,344; *p* = 0.006) were strongly associated with the presence of all three symptoms in multivariate analyses (see Table 2).

**Table 2:** Association of symptom cluster (arthralgia, fatigue, and insomnia) with inflammatory biomarkers

	Univariate analysis		Multivariate analysis	
	Coefficient (95 % CI)	*p* value	Coefficient (95 % CI)	*p* value
CRP	117.3 (49.3–185.3)	0.001	93.1 (25.1–161.1)	0.008
Eotaxin	87.3 (40.8–133.7)	<0.001	79.9 (32.5–127.2)	0.001
MCP-1	178 (63–293.2)	0.003	151.2 (32.7–269.8)	0.013
VDBP	21,530.8 (8088.4–34,973.2)	0.002	19,422 (5500.5–33,344)	0.006

Data shown are results of multivariate linear regression analyses controlled for race, body mass index, chemotherapy status, non-steroidal anti-inflammatory drug use, and age

*CI* confidence interval, *CRP* C-reactive protein, *MCP-1* monocyte chemoattractant protein 1, *VDBP* vitamin D–binding protein

## Discussion

Arthralgia is a common side effect among women taking AIs and is associated with premature discontinuation of therapy [1]. Non-adherence to a 5-year regimen of AIs is associated with increased mortality [2]. Unfortunately, current understanding of this toxicity is limited. We found that moderate to severe arthralgia is associated with specific serum markers of inflammation (elevated CRP, eotaxin, MCP-1, and VDBP) among women taking AIs. We also found that the simultaneous experience of arthralgia, fatigue, and insomnia was associated with elevated serum biomarker concentrations. These data suggest that inflammation may be a shared mechanism of these toxicities.

Our findings are consistent with prior research tying estrogen deprivation to inflammation. Estrogen α- and β-receptors in the nucleus alter gene expression of various inflammatory biomarkers, leading to immunomodulation [5]. Indeed, estrogen supplementation seems to ameliorate certain autoimmune conditions [4], and women going through menopause can experience an exacerbation of inflammatory conditions, such as fibromyalgia and rheumatoid arthritis [7, 8].

On the basis of this biological plausibility, others have evaluated the contribution of inflammation to AI-associated arthralgia. In a genome-wide association study, Ingle et al. identified a series of SNPs that were correlated with the presence of AI-associated arthralgia [11]. These SNPs centered on T-cell leukemia/lymphoma protein 1A (TCL1A), an inflammatory protein whose activity is modulated by estrogen levels. The SNP variations associated with AI-associated arthralgia resulted in TCL1A levels that were more sensitive to estrogen variation. Researchers in two prior studies have explored the association of serum inflammatory biomarkers with AI-associated arthralgia [12, 13]. Although neither of these studies revealed an association between arthralgia and systemic inflammation, both were limited by small sample sizes.

Our research found that arthralgia is significantly associated with fatigue and insomnia. Among those experiencing moderate to severe arthralgia, 88.4 % also had fatigue and 83.7 % also had insomnia. Bower et al. have evaluated the association of inflammatory biomarkers with fatigue both during [26] and after primary treatment [15] for breast cancer. Consistent with our findings, they noted that CRP elevations were associated with fatigue [26]. On a molecular level, estrogen blocks the expression of IL-6, which is the principal stimulatory molecule for the secretion of CRP [5, 6]. It thus may not be surprising that patients have elevated CRP levels during treatment for breast cancer, when estrogen levels fall significantly. Our data suggest that these symptoms often coexist and that inflammation may underlie the mechanism of these common symptoms; therefore, interventions targeting inflammation may play a role in addressing these symptoms simultaneously.

Our identification of VDBP, eotaxin, and MCP-1 as relevant inflammatory biomarkers for AI-related symptom burden is novel. VDBP is the primary binding protein for vitamin D, but it is also an acute-phase reactant subject to significant genetic variability [27–31]. VDBP is thus a particularly intriguing mechanistic molecule, given accumulating evidence that vitamin D may have an important role in a wide variety of disease states [32]. Indeed, high-dose vitamin D supplementation was associated with a decreased incidence of AI-associated arthralgia in one study [33]. Eotaxin and MCP-1 are chemokines responsible for the recruitment of inflammatory cells to sites of injury [34, 35]. Elevated eotaxin and MCP-1 concentrations have been seen in patients with fibromyalgia [36], another condition characterized by joint pain, insomnia, and fatigue [37].

Our study has a number of important limitations. First, it was cross-sectional in nature. As such, we are unable to establish the causal relationship between symptom burden and inflammatory biomarkers. Further prospective work is needed to clarify whether systemic inflammation leads to symptoms or if the persistent symptoms cause inflammation. Second, we examined numerous biomarkers in this study, and there is a risk of false-positive discovery. We adjusted our primary analysis using Bonferroni corrections for those biomarkers that demonstrated variability in our sample. A future replication study is needed to verify the results identified in our study. Third, our biomarker sampling was non-fasting, and we did not have complete data on comorbid conditions or other medications patients may have used, including statins and bisphosphonates. As described above, concurrent autoimmune conditions may also affect biomarker concentrations [36]. Future studies should control for these variables.

In spite of these limitations, this study is, to the best of our knowledge, the largest study to date evaluating the association of patient-reported symptoms with inflammatory biomarkers. We demonstrated that patient-reported arthralgia and co-morbid fatigue and insomnia are associated with elevated serum inflammatory biomarkers among women taking AIs. A better understanding of this novel finding will allow clinicians to develop strategies to manage these symptoms to improve quality of life, adherence to AI therapies, and potentially survival outcomes for women with breast cancer.

## Conclusions

Patient-reported arthralgia and co-morbid fatigue and insomnia were associated with elevated CRP, eotaxin, MCP-1, and VDBP among a group of breast cancer survivors taking AIs. A better understanding of symptomatic causes of distress will allow clinicians to develop strategies to manage these symptoms to improve quality of life, adherence to therapy, and potentially survival outcomes for women with breast cancer.

## Additional files

Additional file 1: Table S1. Pairwise correlation between inflammatory biomarkers. Additional file 2: Tables S2–S4. Two-sided *t* tests of inflammatory biomarker concentrations as a function of symptoms.

## Abbreviations

AI: Aromatase inhibitor
BFI: Brief Fatigue Inventory
BMI: Body mass index
BPI: Brief Pain Inventory
CI: Confidence interval
CRP: C-reactive protein
IFN: Interferon
IL: Interleukin
LMP: Last menstrual period
MCP-1: Monocyte chemoattractant protein 1
MIG: Monokine induced by interferon gamma
MIP: Macrophage inflammatory protein
NSAID: Non-steroidal anti-inflammatory drug
RANTES: Regulated on activation, normal T expressed and secreted protein
SD: Standard deviation
SNP: Single-nucleotide polymorphism
TCL1A: T-cell leukemia/lymphoma protein 1A
VDBP: Vitamin D–binding protein
VEGF: Vascular endothelial growth factor
