# Supplementary material for: Arthralgia among women taking aromatase inhibitors: is there a shared inflammatory mechanism with co-morbid fatigue and insomnia?
Source: Breast Cancer Res. 2015 Jun 28;17(1):89. doi: 10.1186/s13058-015-0599-7 (PMC4504449; doi:10.1186/s13058-015-0599-7)
Supplement: Additional file 1: Table S1. — Pairwise correlation between inflammatory biomarkers. [file 13058_2015_599_MOESM1_ESM.docx]

Table S1: Pairwise Correlation between Inflammatory Biomarkers

|  | **B2M** | **CRP** | **EGF** | **Eotaxin** | **G-CSF** | **Haptoglobin** | **IL-12 p40.p70** | **IL-13** | **IL-1RA** | **IL-2R** | **IP-10** | MCP-1 | **MIG** | **VDBP** |
| --- | --- | --- | --- | --- | --- | --- | --- | --- | --- | --- | --- | --- | --- | --- |
| **B2M** | **1.0** | 0.4 | 0.1 | 0.1 | 0.2 | 0.3 | 0.2 | 0.2 | 0.2 | 0.2 | 0.2 | 0.1 | 0.2 | 0.4 |
| **CRP** | 0.4 | **1.0** | 0.1 | 0.1 | 0.2 | 0.4 | 0.2 | 0.2 | 0.1 | 0.2 | 0.2 | 0.1 | 0.1 | 0.5 |
| **EGF** | 0.1 | 0.1 | **1.0** | 0.3 | 0.5 | 0.1 | 0.5 | 0.4 | 0.5 | 0.5 | 0.2 | 0.2 | 0.2 | 0.2 |
| **Eotaxin** | 0.1 | 0.1 | 0.3 | **1.0** | 0.3 | 0.1 | 0.3 | 0.4 | 0.3 | 0.2 | 0.1 | 0.7 | 0.2 | 0.3 |
| **G-CSF** | 0.2 | 0.2 | 0.5 | 0.3 | **1.0** | 0.2 | 0.6 | 0.6 | 0.6 | 0.6 | 0.4 | 0.3 | 0.4 | 0.3 |
| **Haptoglobin** | 0.3 | 0.4 | 0.1 | 0.1 | 0.2 | **1.0** | 0.2 | 0.2 | 0.2 | 0.2 | 0.1 | 0.2 | 0.1 | 0.5 |
| **IL-12p40.p70** | 0.2 | 0.2 | 0.5 | 0.3 | 0.6 | 0.2 | **1.0** | 0.5 | 0.9 | 0.7 | 0.7 | 0.3 | 0.7 | 0.4 |
| **IL-13** | 0.2 | 0.2 | 0.4 | 0.4 | 0.6 | 0.2 | 0.5 | **1.0** | 0.5 | 0.4 | 0.3 | 0.4 | 0.3 | 0.4 |
| **IL-1RA** | 0.2 | 0.1 | 0.5 | 0.3 | 0.6 | 0.2 | 0.9 | 0.5 | **1.0** | 0.7 | 0.4 | 0.3 | 0.5 | 0.4 |
| **IL-2R** | 0.2 | 0.2 | 0.5 | 0.2 | 0.6 | 0.2 | 0.7 | 0.4 | 0.7 | **1.0** | 0.4 | 0.3 | 0.6 | 0.3 |
| **IP-10** | 0.2 | 0.2 | 0.2 | 0.1 | 0.4 | 0.1 | 0.7 | 0.3 | 0.4 | 0.4 | **1.0** | 0.2 | 0.7 | 0.2 |
| **MCP-1** | 0.1 | 0.1 | 0.2 | 0.7 | 0.3 | 0.2 | 0.3 | 0.4 | 0.3 | 0.3 | 0.2 | **1.0** | 0.2 | 0.2 |
| **MIG** | 0.2 | 0.1 | 0.2 | 0.2 | 0.4 | 0.1 | 0.7 | 0.3 | 0.5 | 0.6 | 0.7 | 0.2 | **1.0** | 0.2 |
| **VDBP** | 0.4 | 0.5 | 0.2 | 0.3 | 0.3 | 0.5 | 0.4 | 0.4 | 0.4 | 0.3 | 0.2 | 0.2 | 0.2 | **1.0** |

Abbreviations: B2M: Beta 2 Microglobulin CRP: C Reactive Protein, EGF: Epidermal Growth Factor, G-CSF: Granulocyte Colony Stimulating Factor, MCP-1: Monocyte Chemotactic Protein 1, MIG: Monokine induced by Gamma Interferon, VDBP: Vitamin D Binding Protein,
