# Supplementary material for: Arthralgia among women taking aromatase inhibitors: is there a shared inflammatory mechanism with co-morbid fatigue and insomnia?
Source: Breast Cancer Res. 2015 Jun 28;17(1):89. doi: 10.1186/s13058-015-0599-7 (PMC4504449; doi:10.1186/s13058-015-0599-7)
Supplement: Additional file 2: Tables S2–S4. — Two-sided t tests of inflammatory biomarker concentrations as a function of symptoms. [file 13058_2015_599_MOESM2_ESM.docx]

Table S2

Two sided t tests of Inflammatory Biomarker Concentrations as a Function of Arthralgia

|  | Moderate to Severe  Arthralgia Mean  Concentration (SD) | No Arthralgia  Mean  Concentration (SD) | p value |
| --- | --- | --- | --- |
| CRP (ng/mL) | 189 (304.3) | 92.5 (135.8) | 0.0026 |
| EGF (pg/mL) | 80.7 (49.5) | 82 (65.3) | 0.9 |
| Eotaxin (pg/mL) | 243.9 (179.7) | 161.7 (105.9) | 0.0002 |
| G-CSF (pg/mL) | 84.7 (60.4) | 86.5 (57.1) | 0.85 |
| Haptoglobin (ng/mL) | 1359400 (818105.2) | 1251127 (748158.6) | 0.41 |
| IL-12 p40/p70 (pg/mL) | 707.1 (394.7) | 617.7 (323.9) | 0.13 |
| IL-13 (pg/mL) | 83.4 (64.6) | 69.4 (57.3) | 0.17 |
| IL-1Ra (pg/mL) | 3478.5 (1324.1) | 3239.3 (1456.5) | 0.33 |
| IL-2R (pg/mL) | 521.9 (275) | 492.3 (300.5) | 0.56 |
| IP-10 (pg/mL) | 116.8 (148.5) | 93.1 (67.3) | 0.13 |
| MCP-1 (pg/mL) | 873.6 (388.2) | 704.1 (285.3) | 0.0017 |
| MIG (pg/mL) | 148.8 (163.1) | 140 (176.5) | 0.77 |
| VDBP (ng/mL) | 115100.7 (44531) | 93389.3 (33440.4) | 0.0006 |
| β2 Microglobulin (ng/mL) | 1633.4 (644.3) | 1607.4 (895.3) | 0.86 |

Abbreviations: CRP: C Reactive Protein, EGF: Epidermal Growth Factor, G-CSF: Granulocyte Colony Stimulating Factor, MCP-1: Monocyte Chemoattractant Protein 1, MIG: Monokine Induced by Gamma Interferon, VDBP: Vitamin D Binding Protein

Table S3

Two sided t tests of Inflammatory Biomarker Concentrations as a Function of Fatigue

|  | Moderate to Severe  Fatigue Mean  Concentration (SD) | No Fatigue  Mean  Concentration (SD) | p value |
| --- | --- | --- | --- |
| CRP (ng/mL) | 145.9 (228.9) | 89.6 (149.4) | 0.04 |
| EGF (pg/mL) | 81.3 (59.1) | 82 (64.5) | 0.94 |
| Eotaxin (pg/mL) | 209.9 (162.6) | 157.4 (93.7) | 0.004 |
| G-CSF (pg/mL) | 90.1 (59.8) | 83.2 (56.2) | 0.4 |
| Haptoglobin (ng/mL) | 1307655 (808051.7) | 1250342 (731410.8) | 0.6 |
| IL-12 p40/p70 (pg/mL) | 666.2 (328.2) | 615.8 (349.9) | 0.3 |
| IL-13 (pg/mL) | 86.5 (64.5) | 62.3 (52.8) | 0.004 |
| IL-1Ra (pg/mL) | 3420.8 (1307.2) | 3197.3 (1509.1) | 0.27 |
| IL-2R (pg/mL) | 513.7 (260.6) | 487.9 (317.6) | 0.54 |
| IP-10 (pg/mL) | 99.3 (68.6) | 97.3 (104.2) | 0.87 |
| MCP-1 (pg/mL) | 793.8 (364.8) | 702 (272.8) | 0.04 |
| MIG (pg/mL) | 128.4 (111.5) | 151.2 (206.1) | 0.36 |
| VDBP (ng/mL) | 106081.8 (39171) | 92265.7 (34493.6) | 0.009 |
| β2 Microglobulin (ng/mL) | 1715.7 (984.7) | 1539.8 (728.6) | 0.15 |

Abbreviations: CRP: C Reactive Protein, EGF: Epidermal Growth Factor, G-CSF: Granulocyte Colony Stimulating Factor, MCP-1: Monocyte Chemoattractant Protein 1, MIG: Monokine Induced by Gamma Interferon, VDBP: Vitamin D Binding Protein

Table S4

Two sided t tests of Inflammatory Biomarker Concentrations as a Function of Insomnia

|  | Moderate to Severe  Insomnia  Mean Concentration (SD) | No Insomnia  Mean  Concentration (SD) | p value |
| --- | --- | --- | --- |
| CRP (ng/mL) | 148.9 (253) | 95.2 (143.6) | 0.06 |
| EGF (pg/mL) | 79 (55.5) | 83.1 (65.3) | 0.66 |
| Eotaxin (pg/mL) | 219.8 (168.4) | 159 (99.2( | 0.001 |
| G-CSF (pg/mL) | 88.9 (61.2) | 84.7 (56) | 0.63 |
| Haptoglobin (ng/mL) | 1345084 (810497) | 1238984 (738526) | 0.35 |
| IL-12 p40/p70 (pg/mL) | 644.2 (353.6) | 633 (336) | 0.82 |
| IL-13 (pg/mL) | 85.9 (65.9) | 65.6 (54.3) | 0.02 |
| IL-1Ra (pg/mL) | 3248.6 (1330.9) | 3310.9 (1480.3) | 0.77 |
| IL-2R (pg/mL) | 488 (271.6) | 503.9 (306.6) | 0.72 |
| IP-10 (pg/mL) | 98.4 (72.6) | 98 (99) | 0.97 |
| MCP-1 (pg/mL) | 825.4 (376.7) | 697.9 (274.1) | 0.006 |
| MIG (pg/mL) | 133.5 (125.9) | 145.8 (192.7) | 0.64 |
| VDBP (ng/mL) | 107588.4 (43117) | 93257.8 (32785) | 0.009 |
| β2 Microglobulin (ng/mL) | 1582.7 (547.7) | 1628 (962.8) | 0.72 |

Abbreviations: CRP: C Reactive Protein, EGF: Epidermal Growth Factor, G-CSF: Granulocyte Colony Stimulating Factor, MCP-1: Monocyte Chemoattractant Protein 1, MIG: Monokine Induced by Gamma Interferon, VDBP: Vitamin D Binding Protein
